# L-Carnitine: An Antioxidant Remedy for the Survival of Cardiomyocytes under Hyperglycemic Condition

**DOI:** 10.1155/2018/4028297

**Published:** 2018-12-09

**Authors:** Fernanda Vacante, Pamela Senesi, Anna Montesano, Alice Frigerio, Livio Luzi, Ileana Terruzzi

**Affiliations:** ^1^Metabolism Research Center, IRCCS Policlinico San Donato, San Donato Milanese, Milan, Italy; ^2^Department of Biomedical Sciences for Health, Università degli Studi di Milano, Milan, Italy

## Abstract

**Background:**

Metabolic alterations as hyperglycemia and inflammation induce myocardial molecular events enhancing oxidative stress and mitochondrial dysfunction. Those alterations are responsible for a progressive loss of cardiomyocytes, cardiac stem cells, and consequent cardiovascular complications. Currently, there are no effective pharmacological measures to protect the heart from these metabolic modifications, and the development of new therapeutic approaches, focused on improvement of the oxidative stress condition, is pivotal. The protective effects of levocarnitine (LC) in patients with ischemic heart disease are related to the attenuation of oxidative stress, but LC mechanisms have yet to be fully understood.

**Objective:**

The aim of this work was to investigate LC's role in oxidative stress condition, on ROS production and mitochondrial detoxifying function in H9c2 rat cardiomyocytes during hyperglycemia.

**Methods:**

H9c2 cells in the hyperglycemic state (25 mmol/L glucose) were exposed to 0.5 or 5 mM LC for 48 and 72 h: LC effects on signaling pathways involved in oxidative stress condition were studied by Western blot and immunofluorescence analysis. To evaluate ROS production, H9c2 cells were exposed to H_2_O_2_ after LC pretreatment.

**Results:**

Our *in vitro* study indicates how LC supplementation might protect cardiomyocytes from oxidative stress-related damage, preventing ROS formation and activating antioxidant signaling pathways in hyperglycemic conditions. In particular, LC promotes STAT3 activation and significantly increases the expression of antioxidant protein SOD2. Hyperglycemic cardiac cells are characterized by impairment in mitochondrial dysfunction and the CaMKII signal: LC promotes CaMKII expression and activation and enhancement of AMPK protein synthesis. Our results suggest that LC might ameliorate metabolic aspects of hyperglycemic cardiac cells. Finally, LC doses herein used did not modify H9c2 growth rate and viability.

**Conclusions:**

Our novel study demonstrates that LC improves the microenvironment damaged by oxidative stress (induced by hyperglycemia), thus proposing this nutraceutical compound as an adjuvant in diabetic cardiac regenerative medicine.

## 1. Introduction

Cardiovascular complications are recognized as the primary cause of mortality in subjects with diabetes mellitus (DM) [1, 2], characterized by hyperglycemia which is determined by a defect of insulin secretion, insulin action, or both [3]. Moreover, DM is associated with inflammation condition.

Chronic hyperglycemia, characterizing overt diabetes, or fluctuant hyperglycemia, present in the prediabetic condition, are responsible for the activation of numerous signaling pathways that exacerbate the systemic inflammation and lead to the development of diabetic complications [4, 5]. Recent evidence establishes how hyperglycemia is involved in the regulation of sirtuin (SIRT) transcription factors. The deregulation of SIRT expression is strictly correlated with the progression of inflammation and atherosclerotic disease. In particular, Balestrieri et al. demonstrated that SIRT6 protein expression is downregulated in atherosclerotic plaques of diabetics, and this defect is linked to the chronic oxidative stress condition [6]. Those evidences indicate that chronic inflammation, oxidative stress condition and predisposition to ischemic heart disease are higher in patients with DM than in nondiabetics [7, 8].

Traditional therapeutic approaches as well as innovative promising strategies [7] (i.e., stem/progenitor cell therapy, existing cardiomyocyte proliferation, and reprogramming noncardiac cells) are limited in patients with DM [8–11]. Periprocedural intensive glycemic control, during early percutaneous coronary intervention in diabetic patients, was shown to improve myocardial protection by increasing SIRT1 expression, endothelial progenitor cell number, and their capability to differentiate in mature cardiomyocytes [12]. Hyperglycemia in diabetic subjects is the major factor responsible for the failure of regenerative myocardial therapeutic strategies. Recent data indicate that the overproduction of reactive oxygen species (ROS) and the oxidative stress condition are the main causes involved in diabetic cardiac injury and in the lack of success in cardiac regenerative therapies [13–17]. Hyperglycemia enhances ROS production impairing cardiac microenvironment and regeneration capacity [16, 17]. In particular, several publications showed that the hyperglycemic and oxidative microenvironment induces mitochondrial abnormalities and cellular damage, eventually leading to senescence and apoptosis of cardiac progenitors [18, 19].

Thus, to optimize regenerative strategies for diabetic patients, the development of new therapeutic approaches focused on the reduction of oxidative stress condition is fundamental.

Noteworthy, in recent years, L-carnitine (LC) has been proposed as a nutraceutical integrator in the treatment of numerous cardiac syndromes, including coronary disease, atherosclerosis, and toxic myocardial injury [20–22]. It is well established that LC, facilitating transport of long-chain fatty acids into the mitochondrial matrix, plays an important role in supporting cardiac energy homeostasis [23–25]. Most importantly, some studies successfully showed LC's ability to reduce oxidative stress, hypoxic cellular damage, and apoptosis of cardiac cells in normal glycemic condition [26, 27]. In particular, Mao et al. have recently demonstrated that LC pretreatment ameliorated cellular damage induced by H_2_O_2_ in H9c2 rat cardiomyocytes, enhancing mitochondrial function [28].

LC effects on cardiac metabolism and function have been demonstrated under a variety of clinical conditions [29, 30], while LC antioxidative effects in hyperglycemic cardiomyocites were not investigated, yet.

The aim of this study is to investigate *in vitro* LC ability to counteract oxidative stress condition, modifying ROS production and promoting mitochondrial detoxifying function in H9c2 rat cardiomyocytes under hyperglycemic condition (25 mmol/L glucose). The goal is to identify in LC a novel adjuvant agent in cell therapy able to ameliorate the microenvironment of the hyperglycemic heart, thereby supporting cardiac progenitor cell proliferation and differentiation.

## 2. Materials and Methods

### 2.1. Chemicals and Reagents

All utilized reagents were obtained from Sigma Chemical Co. (St. Louis, MO, USA). Primary antibodies against GAPDH (FL-335), p-CaMKII *α* (22B1), CaMKII (M-176), AMPK *α*1/2 (H-300), SOD2 (FL-222), p21 (C-19), peroxidase-conjugated secondary antibodies for Western blot analysis, and FITC-conjugated antibodies for immunofluorescence study were purchased from Santa Cruz Biotechnology (Santa Cruz, CA, USA). STAT3 (124H6) and p-STAT3 (Ser727) were purchased from Cell Signaling Technology (Danvers, MA, USA).

The CellROX® Oxidative Stress Reagent kit (C10443) was purchased from Thermo Fisher Scientific, Life Technologies Italia (Monza, Italy).

### 2.2. Cell Line and Culture Conditions

**Figure 1:**
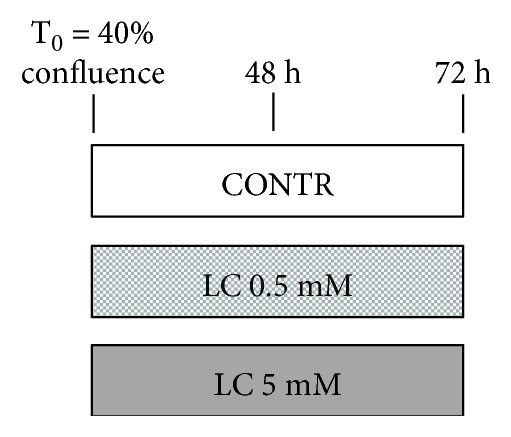
Scheme of treatment. Upon reaching 40% confluence, H9c2 cells were treated for 48 and 72 h with 0.5 or 5 mM LC.

The American Type Culture Collection (ATCC, Manassas, VA, USA) offered the cardiomyoblast H9c2 cell line of rat embryo. The H9c2 cell line was regularly cultured in Dulbecco's modified Eagle's medium (DMEM, Gibco, Grand Island, NY, USA) containing 25 mmol/L glucose, 100 *μ*g/mL streptomycin (Sigma-Aldrich, St. Louis, MO, USA), 100 U/mL penicillin, and 10% fetal bovine serum (FBS, Gibco, Grand Island, NY, USA) in 75 cm^2^ tissue culture flasks and then incubated at 37°C by adopting 5% CO_2_. H9c2 cells exposed to high glucose (25 mmol/L) represent a validated *in vitro* model in order to mimic cardiac hyperglycemic/diabetic condition [31, 32]. Cells were fed every day; following literature indications, H9c2 cells were split when reaching 70–80% confluence in order to prevent the loss of the differentiation potential [28, 32]. Preliminarily, we evaluated the effective concentration of LC based on our previous work performed on skeletal myoblasts and literature evidence (data not shown) [28, 33]. We identified the correct LC concentrations as the lower concentrations that activated STAT3 signaling. Then, H9c2 cells in the active proliferation phase were treated with 0.5 or 5 mM LC as indicated in Figure 1.

### 2.3. Growth Curve and Cell Viability Test

H9c2 cells were plated on 60 mm × 15 mm culture dishes at 20% confluence and grown in DMEM. After an overnight phase, the cells were treated or not with 0.5 or 5 mM LC. At 24, 48, and 72 h after treatments, cells were trypsinized and stained with trypan blue and were counted using hemocytometer. The average values for each single day were used to plot a growth curve. Cell viability was calculated by dividing the unstained viable cell count by the total cell count. In addition, morphological changes were observed daily by phase contrast microscopy.

### 2.4. Western Blot Analysis

Western blot analysis was performed as described previously [34]. Cell lysates were prepared using RIPA buffer implemented with protease inhibitors. 30 *μ*g of proteins was separated by SDS-polyacrylamide gel electrophoreses (SDS-PAGE) and electrophoretically transferred to nitrocellulose membranes (Protran®, Whatman® Schleicher & Schuell). The blots were then blocked and incubated with specific primary antibodies, followed by incubation with anti-species-specific secondary antibodies. To confirm equal protein loading per sample, we used GAPDH protein as housekeeping protein. Finally, detection of specific proteins was performed by enhanced chemoluminescence reagent (Western Lightning ECL Pro, Perkin Elmer). Quantitative measurement of immunoreactive band intensities was performed by densitometric analysis using the Scion Image software (Scion Corporation, Frederick, MD, USA). Data were then converted into fold changes (FC) of the control.

### 2.5. Immunofluorescence Studies

H9c2 cells were grown on coverslips with or without LC 0.5 or 5 mM LC. After 48 and 72 h of treatment, cells were washed 3 times with PBS, then fixed in prepared 4% paraformaldehyde for 20 minutes. The cells on the coverslips were washed with PBS and incubated for 30 minutes at room temperature with 1% bovine serum albumin in PBS with 0.2% Triton X-100. At that point, H9c2 cells were incubated with primary antibodies for 120 minutes. To detect the primary antibody, binding site cells were washed three times in PBS and followed by incubation with specific antibodies FITC-conjugated for 90 minutes. Nuclei were revealed with DAPI staining. Coverslips with cells were mounted and observed using Nikon Eclipse 50I microscopy. The images were captured using Nis-Elements D 4.00 software. Immunofluorescence signals were estimated using the ImageJ program (http://imagej.nih.gov/ij/). Data were displayed and analyzed using Adobe Photoshop CS4®.

Automated quantification on the immunofluorescence signal was performed by using the ImageJ program (http://imagej.nih.gov/ij/) [34]. For each analyzed protein, the quantified signal was normalized for the total nuclei number.

### 2.6. Intracellular ROS Determination in H9c2 Cells after H_2_O_2_ Injury

**Figure 2:**
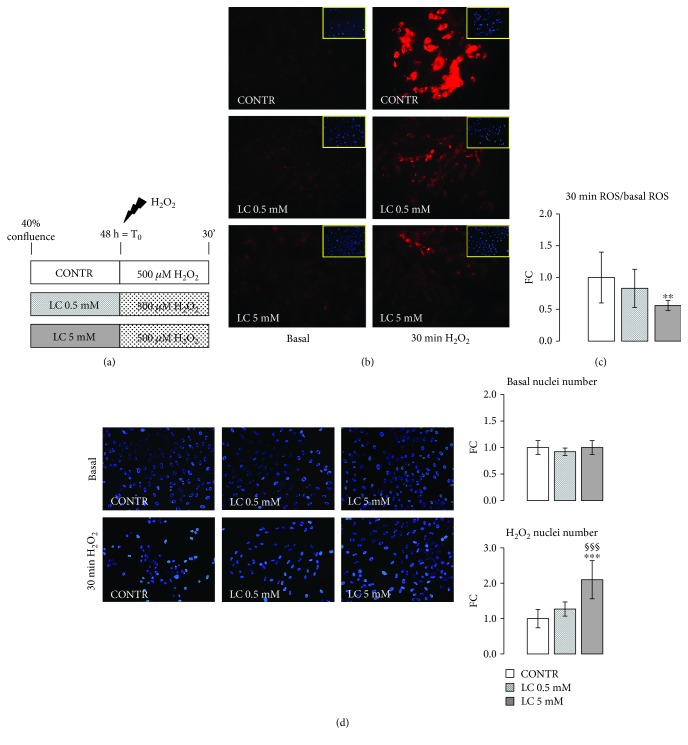
Attenuation of ROS production induced by H_2_O_2_ in LC-pretreated H9c2 cells. (a) Scheme of treatment: H9c2 cardiomyocytes were exposed to a stressful insult with 500 *μ*M of H_2_O_2_ after pretreatment for 48 h with 0.5 or 5 mM LC. (b) Using the CellROX reagent assay, LC action was evaluated under oxidative stress conditions. The pretreatment with 0.5 or 5 mM LC causes a reduction in ROS production after stimulus with 500 *μ*M of H_2_O_2_. (c) Quantification of ROS production. The quantified signal was normalized for the total nuclei number. From the DAPI images, it was possible to observe that the cells pretreated with both doses of LC show higher survival compared to the control cells. (d) Quantification of nuclei number pre- and after H_2_O_2_ injury in LC-pretreated H9c2 cells. Data are expressed as fold changes (FC) of mean ± SD. Significance: ^∗∗^*p* ≤ 0.01 vs. CONTR.

Intracellular ROS levels were valued using the CellROX® Oxidative Stress Reagent kit. Briefly, H9c2 cells were pretreated with 0.5 or 5 mM LC for 48 h, and then they were exposed to 500 *μ*M H_2_O_2_ for 30 min. At the end of the H_2_0_2_ injury, the fluorogenic probe of the kit was added (Figure 2(a)). CellROX® Oxidative Stress Reagents are fluorogenic probes designed to reliably measure ROS in live cells. The cell-permeable reagents are nonfluorescent or very faintly fluorescent while in a reduced state and during oxidation exhibit a strong fluorogenic signal. The CellROX® Orange Reagent signal is localized in the cytoplasm.

The images were captured using Nis-Elements D 4.00 software. Data were displayed and analyzed using Adobe Photoshop CS4®.

Automated quantification on the immunofluorescence signal was performed by using the ImageJ program (http://imagej.nih.gov/ij/) [35]. For each analyzed protein, the quantified signal was normalized for the total nuclei number.

### 2.7. Statistical Analysis

All experiments were performed three times. The data are expressed as the means ± standard deviation, and statistical comparisons were performed with specific statistical packages (Prism v 7.00 GraphPad Software, San Diego, CA, USA). Differences were analyzed by one- or two-way analysis of variance (ANOVA) followed by Tukey's multiple comparison post hoc test. *p* < 0.05 was considered statistically significant.

## 3. Results

### 3.1. Attenuation of ROS Production Induced by H_2_O_2_ in LC Pretreated H9c2 Cells

The diabetic heart usually has ROS levels that exceed normal quantities, and ROS overproduction likely contributes to cardiomyopathy [15, 36, 37]. After stimulus with 500 *μ*M H_2_O_2_, 48 h pretreatment with 0.5 or 5 mM LC markedly reduced ROS levels with respect to the CONTR condition (Figures 2(b) and 2(c)). Furthermore, DAPI images showed that pretreated H9c2 cells with LC, in particular with 5 mM LC, showed a higher number of nuclei than the control condition (Figures 2(b) and 2(d)): These results suggested that LC pretreatment improved cellular survival after H_2_O_2_ injury.

### 3.2. LC Improves Antioxidant Response in H9c2 Cells under Hyperglycemic Condition

**Figure 3:**
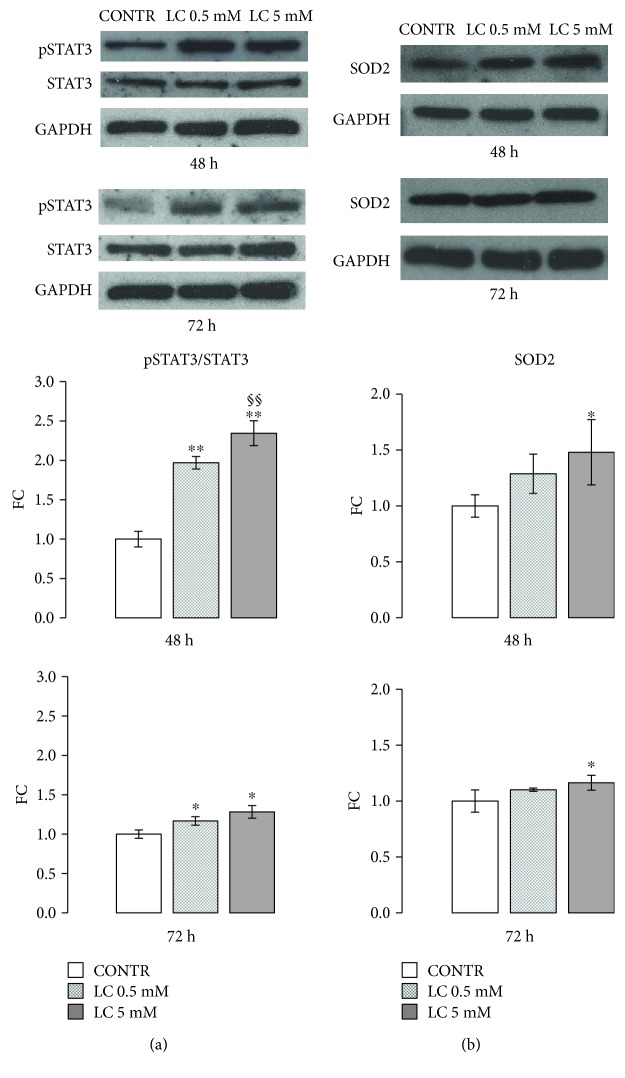
LC improves antioxidant response in H9c2 cells under hyperglycemic condition. (a) Representative Western blot and relevant quantification: LC stimuli for 48 and 72 h significantly enhanced STAT3 activation (ratio pSTAT3/STAT3). (b) Western blot analysis and relevant quantification: 5 mM LC improved SOD2 protein content in H9c2 cells after 48 and 72 h of treatment with respect to control cells. Data are expressed as fold changes (FC) of mean ± SD. Significance: ^∗^*p* ≤ 0.05 vs. CONTR; ^∗∗^*p* ≤ 0.01 vs. CONTR; ^§§^*p* ≤ 0.01 vs. LC 0.5 mM.

Signal transducer and activator of transcription 3 (STAT3) activation thought phosphorylation on Ser^727^ is an important protective mechanism to prevent ROS generation in the setting of oxidative stress [38–40]. Under normoxic conditions, the treatment with 0.5 or 5 mM LC for 48 and 72 h significantly enhanced myocardial STAT3 phosphorylation on Ser^727^ (Figure 3(a)).

Moreover, after 48 and 72 h of treatment, exposure of cells to 0.5 mM LC caused rapid increase of SOD2, a well-known antioxidant enzyme [41]. This effect was significantly augmented with the 5 mM LC dose (Figure 3(b)).

### 3.3. LC Downregulation of CaMKII Pathway

**Figure 4:**
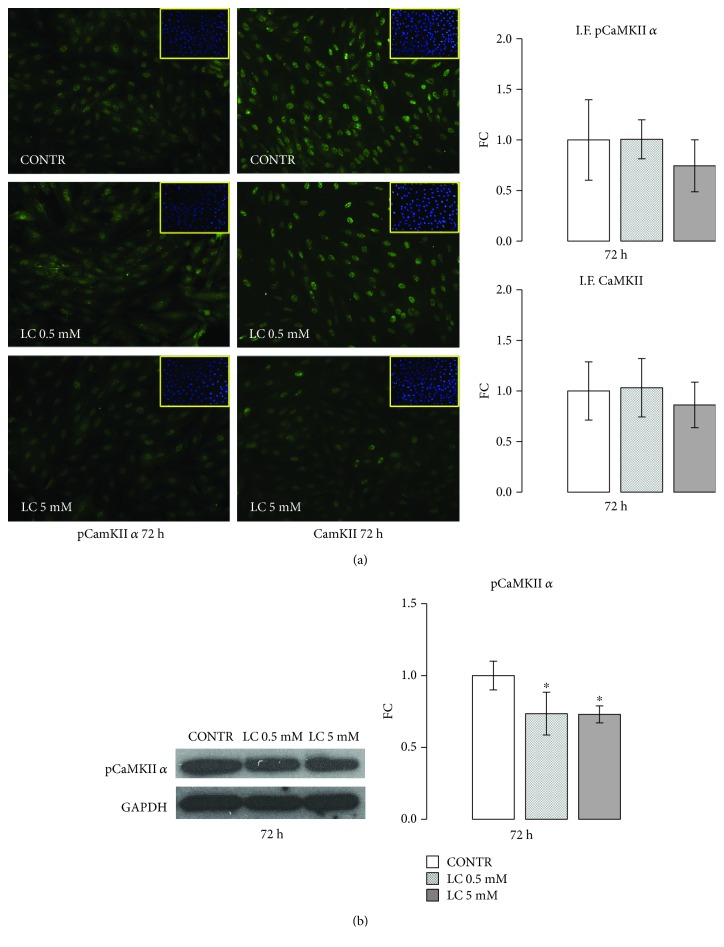
LC downregulation of CaMKII pathway. (a) LC treatments decreased CaMKII-positive cell number and its *α* phosphorylated form after 72 h of stimuli: this effect was more marked for the 5 mM LC dose. The quantified signal was normalized for the total nuclei number. (b) Representative Western blot and relevant quantification: LC stimuli for 72 h promote CaMKII *α* isoform activation. Data are expressed as fold changes (FC) of mean ± SD. Significance: ^∗^*p* ≤ 0.05 vs. CONTR.

Ca^2+^/calmodulin-dependent protein kinase II (CaMKII), a multifunctional serine/threonine protein kinase, is implicated in the pathogenesis of cardiac diseases [42, 43] promoting ROS overproduction [44, 45]. As shown by immunofluorescence analysis (Figure 4(a)), compared with untreated H9c2 cells, LC treatments decreased CaMKII and pCaMKII-positive cell numbers after 72 h of stimuli: immunofluorescence quantification shows a greater effect with the 5 mM LC dose. Furthermore, after 72 h, either 0.5 or 5 mM doses of LC significantly decreased the phosphorylation of CaMKII *α* isoform, as shown by Western blot analysis (Figure 4(b)).

### 3.4. LC Action on AMPK Protein Expression

**Figure 5:**
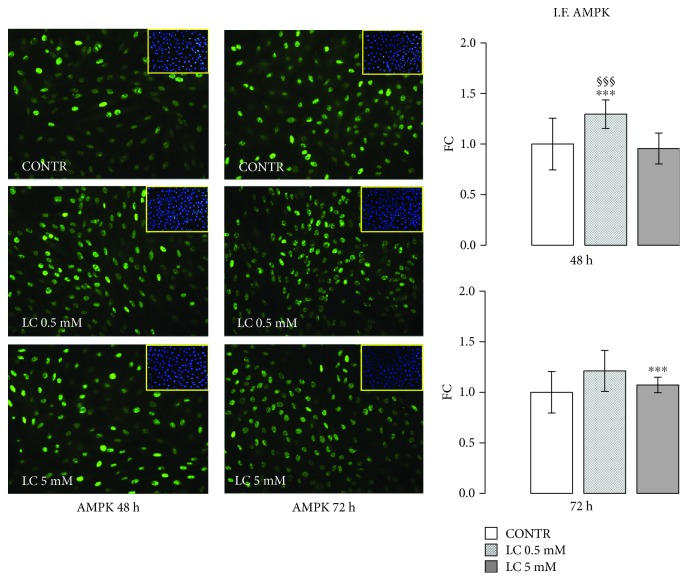
LC action on AMPK protein expression. Immunofluorescence assay and relevant quantification showed an increase in the number of AMPK-positive cells in LC condition at 48 and 72 h. The quantified signal was normalized for the total nuclei number. Data are expressed as fold changes (FC) of mean ± SD. Significance: ^∗∗∗^*p* ≤ 0.001 vs. CONTR; ^§§§^*p* ≤ 0.001 vs. LC 5 mM.

5′-AMP-activated kinase (AMPK) has become a strategic cellular target for the cure of cardiovascular disease correlated with DM [46–48]: it is likely that AMPK activity in the diabetic heart may ameliorate cardiac function. After LC stimuli, immunofluorescence for AMPK showed an increase in AMPK-positive cells in a dose- and time-dependent manner (Figure 5).

### 3.5. LC Action on Cardiomyocyte Viability

**Figure 6:**
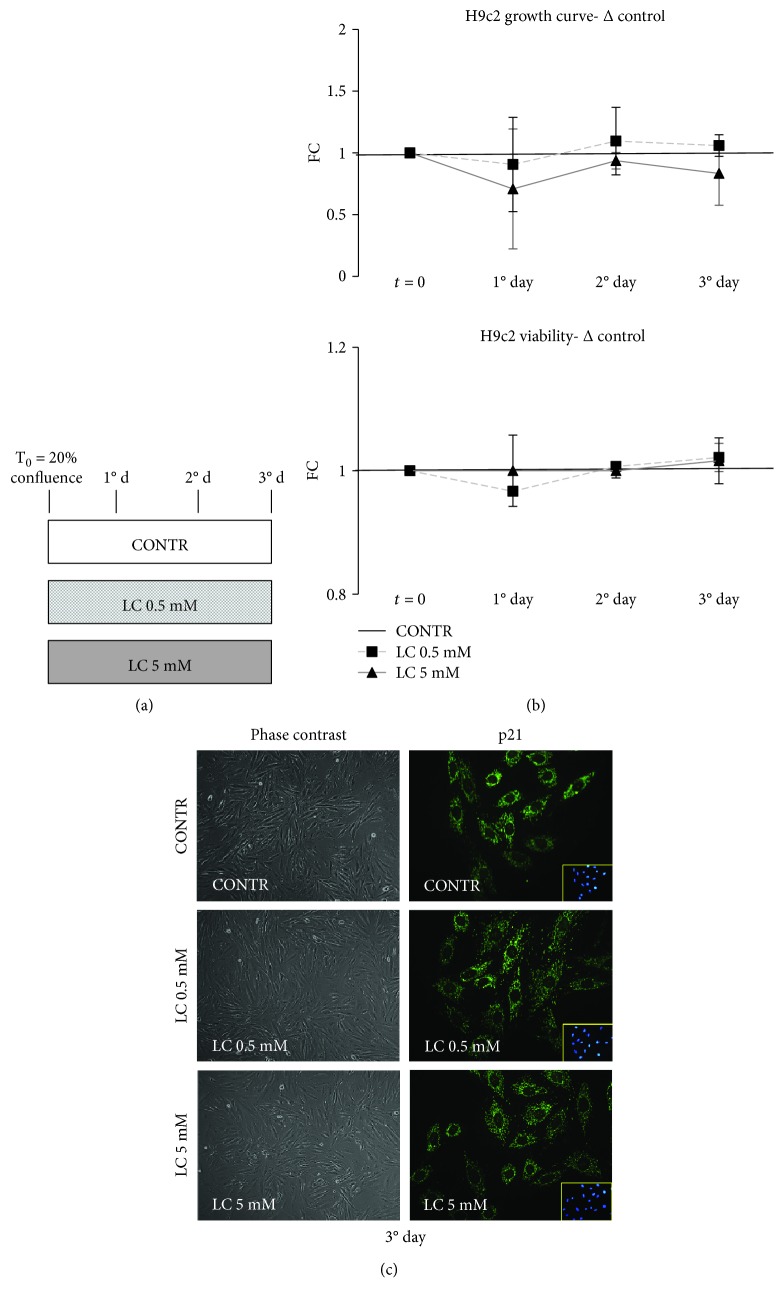
LC action on cardiomyocyte viability. (a) Experimental scheme for growth curve and viability determination. (b) 0.5 or 5 mM LC did not alter the H9c2 proliferative potential. Viability graph shows the absence of cell mortality in all treatment conditions. (c) Phase contrast images show how treatment with 0.5 or 5 mM LC do not modify the morphology of H9c2 cells. Furthermore, there is no p21 translocation from cytoplasm to nuclei following treatment with both doses of LC. Data are expressed as fold changes (FC) of mean ± SD.

An uncontrolled consequence of sustained hyperglycemia is the induction of cardiomyocyte death that causes a loss of contractile units, which declines organ function and provokes hypertrophy of vital cardiomyocytes [49–51]. We investigated viability and morphologic features of H9c2 cells after exposure to 0.5 or 5 mM LC (Figure 6(a)). The growth curve showed that LC treatments did not induce a change of cellular proliferation with respect to untreated control cells (Figure 6(b)). Moreover, the viability graph showed the absence of cell mortality in all treatment conditions (Figure 6(b)). To support these data, phase contrast images, collected at day 3 of the growth curve, confirmed the absence of morphological changes in cells treated with 0.5 or 5 mM LC respect to control (Figure 6(c)).

In opposition to its antiproliferative functions, p21 can also play proproliferative and survival roles when it is localized in the cytosol [51]. Figure 6(c) shows that the protein content of p21 in cardiomyocytes treated with 0.5 or 5 mM LC was superimposable to control cells, and this result highlights that there is not p21 translocation from cytoplasm to nuclei.

## 4. Discussion

In the present study, we demonstrated that LC supplementation significantly decreases ROS production in cardiomyocytes during hyperglycemia (Figure 2). ROS generation induced by high glucose causes apoptosis of cardiac cells and important decrease in growth factor secretion [15, 16, 52–55]. In the heart microenvironment, the oxidative stress induced by hyperglycemia can lead to stem cell senescence which is characterized by the production and secretion of soluble factor SASP (senescence-associated secretory phenotype). Those factors, responsible for the onset of chronic inflammation and oxidative stress, are considered the pathophysiological link between aging and diabetes in cardiovascular diseases [56].

The association hyperglycemia/ROS/cellular senescence represents a major cause of inefficiency of regenerative medicine [9, 10, 56, 57]. Further, clinical trials established that an altered glycemic control is equal to therapeutic failure [13, 14]. Our data point out that LC could counteract oxidative stress in the hyperglycemic condition. Those observations are consistent with several previous works that reported that LC stimuli decrease ROS generation in the skeletal muscle, bone and cardiac cells grown under normoglycemic condition [26, 27, 58, 59]. Furthermore, as shown in Figure 6, both doses of LC did not modify the H9c2 growth rate and did not induce cellular damages.

Studying the mechanism by which LC may modulate ROS formation, we observed an increase in the serine 727 (Ser^727^) phosphorylation of STAT3, after 48 and 72 hours of treatment (Figure 3(a)). Recent evidence stresses the critical role of STAT3 in modulating mitochondrial respiratory chain function and ROS production [39, 40]. Serine 727 (Ser^727^) phosphorylation has a primary part in STAT3 influence on mitochondria: its decrement is associated with the development of cardiac hypertrophy and dilated cardiomyopathy [60]. Moreover, several data showed that STAT3 conserves complex I activity in ischemic condition enhancing cell viability and plays an important role in heart protection from chronic stress induced by hyperglycemia [61]. STAT3 activation promotes the expression of SOD2, the principal enzyme effective in reducing mitochondrial oxidative species [41, 62]. LC treatment significantly increased SOD2 expression in *in vitro* cardiomyocytes, confirming LC capability to counteract oxidative stress in the hyperglycemic condition (Figure 3(b)).

Interestingly, a number of studies have indicated that CaMKII enhances mitochondrial dysfunction, ROS formation, and apoptosis [63], eventually causing cardiomyocyte death both following hyperglycemia [64, 65] and oxidative stress conditions [43, 45, 66].

Hyperglycemic cardiac cells are characterized by a vicious circle between ROS/mitochondrial dysfunction/CaMKII that triggers cellular damages and apoptosis phenomena [42–44]. Taking into consideration that literature evidence, we investigated LC action on CaMKII protein content. As reported in Figure 4, LC treatments reduce CaMKII activation. Remarkably, recent experiments hypothesized that CaMKII activation is related to Ca^2+^ movement from the endoplasmic reticulum to mitochondria.

Recently, LC was shown to promote Ca^2+^ availability which is needed for proliferation and differentiation of human osteoblast-like cells, via a depolarization of L-type calcium channels [59]. Despite the Ca^2+^ supply via the L-type channels being essential to ensure appropriate cardiac cell contraction, an excessive Ca^2+^ influx increases mitochondrial ROS production in cardiomyocytes under oxidative stress. Various authors suggested that cardiac damage, induced by CaMKII activation, is caused by an increase in L-type Ca^2+^ current [67].

LC capability of modulating CaMKII in cardiac cells could make LC a molecule appropriate for impeding the cross-talk between L-type Ca^2+^ channels and ROS production and restoring normal mitochondrial function in cardiac cells. Further investigations are necessary to unravel LC action on cardiac calcium channels.

AMPK represents a strategic target in cardiovascular alterations associated with DM. It is well known that impairment of AMPK activation characterizes hyperglycemic cardiomyocytes. In particular, during hypoxia/reoxygenation (H/R) injury in diabetic patients, AMPK deficiency is associated with an increase in ROS production and apoptosis [46, 47]. As shown in Figure 5, LC stimuli enhanced AMPK protein synthesis, suggesting that LC not only could ameliorate the oxidative microenvironment but also could improve metabolic functions of cardiac cells. With this in mind, future studies could investigate LC's possible action on the protein kinase C (PKC) signaling pathway, implicated in diabetic damage. As known, in hyperglycemic condition, the decrease in AMPK activity is associated with an increase of diacylglycerol (DAG) production that activates PKC and NADPH-oxidase causing an abnormal production of ROS [68, 69]. Then, LC ability to stimulate AMPK activation and to regulate the production of acyl-CoA could counteract hyperinsulinemia-correlated oxidative stress.

Whereas the higher risk of developing atrial fibrillation (AF) in diabetic subjects compared to healthy ones is associated with mitochondrial ROS overproduction, ATP depletion, and abnormal calcium homeostasis [70, 71], the key modulator role shown by LC suggests the potential use of this micronutrient as adjuvant therapy in different cardiac pathologies associated with DM.

In conclusion, LC described effects could be useful in association with other cardioactive drugs. Ranolazine is an antianginal drug with hypoglycemic action used in AF treatment, which was shown to enhance skeletal muscle differentiation [35]. In cardiac regenerative medicine, antioxidative LC action, in association with ranolazine, might lead to the improvement of the hyperglycemic-oxidative microenvironment, prevention of apoptosis, and preservation of cell viability. Equally interesting could be the synergistic effect of LC with allopurinol [72] or the *α*-lipoic acid antioxidant [73] in the prevention and treatment of AF. Remarkable therapeutic perspectives could arise from the association of LC with new hypoglycemic drugs, such as incretins, whose effects on the hyperglycemia control, inflammation, and atherosclerotic plaque progression have recently been demonstrated [74, 75].

Taken together, our results argue that the use of LC as a coadjuvant in therapeutic treatments is very promising, although extensive studies focused on the determination of the effective LC dose and route of administration in humans should be undertaken. Anyway, LC doses used in our study (0.5–5 mM) are compatible with ranges used in humans receiving LC therapy [76, 77].

## 5. Conclusion

The results of this work (Table 1) provide, for the first time, fundamental *in vitro* cellular evidence that treatment with LC could be a potential strategy to improve the cardiac oxidative microenvironment caused by hyperglycemia.

According to the findings of the present study, demonstrating the LC ability to decrease production of ROS, activate STAT3 and AMPK, and downregulate CaMKII, LC could represent a potential adjuvant therapeutic strategy in diabetic cardiac regenerative medicine.

This adjuvant therapy might be recommended for diabetic patients with high risk of cardiac injury as it exerts a cardioprotective effect by restoring the microenvironmental equilibrium by strengthening cardiomyocytes' defense mechanisms through the stimulation of endogenous antioxidants (SOD2).

Further, *in vitro* and *in vivo* studies on this topic are essential to improve the knowledge on the effects of LC in cardiac diseases associated with diabetes and on its potential synergic action with other antioxidative and hypoglycemic agents.

## Figures and Tables

**Table 1:** Summary of LC action on hyperglycemic cardiomyocytes (H9c2).

Hyperglycemic cardiomyocites (H9c2)					
L-Carnitine treatment	ROS	STAT3	CaMKII	AMPK	Cellular growth and vitality
	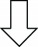	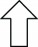	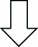	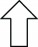	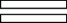

## Data Availability

The authors confirm that the data supporting the findings of this study are openly available within the article.
